# Herpes simplex virus replication compartments: From naked release to recombining together

**DOI:** 10.1371/journal.ppat.1007714

**Published:** 2019-06-03

**Authors:** Oren Kobiler, Matthew D. Weitzman

**Affiliations:** 1 Department of Clinical Microbiology and Immunology, Sackler School of Medicine, Tel Aviv University, Tel Aviv, Israel; 2 Department of Pathology and Laboratory Medicine, Perelman School of Medicine, University of Pennsylvania, Philadelphia, Pennsylvania, United States of America; 3 Division of Protective Immunity, Children’s Hospital of Philadelphia, Philadelphia, Pennsylvania, United States of America; University of Michigan Medical School, UNITED STATES

## Introduction

Amplification of viral genomes is key to successful infection and spread. Herpes simplex virus 1 (HSV-1) is a successful and ubiquitous human virus. HSV-1 acute infection outcomes range from asymptomatic infections to life-threatening encephalitis. Primary infection leads to life-long, persistent, latent infection, which can periodically reactivate to result in characteristic “cold sores.” Herpesviruses are considered ancient viruses that have coevolved with their hosts for a long time, generating complex virus–host interactions [1]. Because HSV replicates in the nucleus, interactions at sites of viral replication determine outcomes of infection, and interactions between viral genomes and the complex nuclear environment contribute to efficient infection.

There are three phases of HSV-1 viral gene expression from the double-stranded DNA genome: immediate early (IE), early (E), and late (L). These are temporally and spatially regulated within the infected cell nucleus. IE proteins are involved in the takeover of gene transcription and countering of intrinsic host defenses. E proteins are mainly involved in viral DNA replication. L genes are activated after the onset of viral DNA replication, and their protein products are involved in assembly and packaging of progeny virions. Although this cascade of gene expression has been well characterized at the population level, the temporal variation among individual infected cells is less well understood, and it is unclear whether all entering genomes follow the entire viral genetic cascade.

Sequestering of viral replication machinery at specific intracellular locations is a common phenomenon observed in virology. This highlights the important evolutionary benefits of viral-induced structures, which serve to both concentrate required proteins and also limit access by detrimental host factors. Like many other viruses, herpesviruses confine their gene expression and replication to distinct intranuclear sites known as replication compartments (RCs). In this short review, we discuss the formation of these structures, their ability to attract viral and cellular proteins, and the impact of interactions between distinct individual RCs.

## What is the origin of each RC?

**Fig 1:**
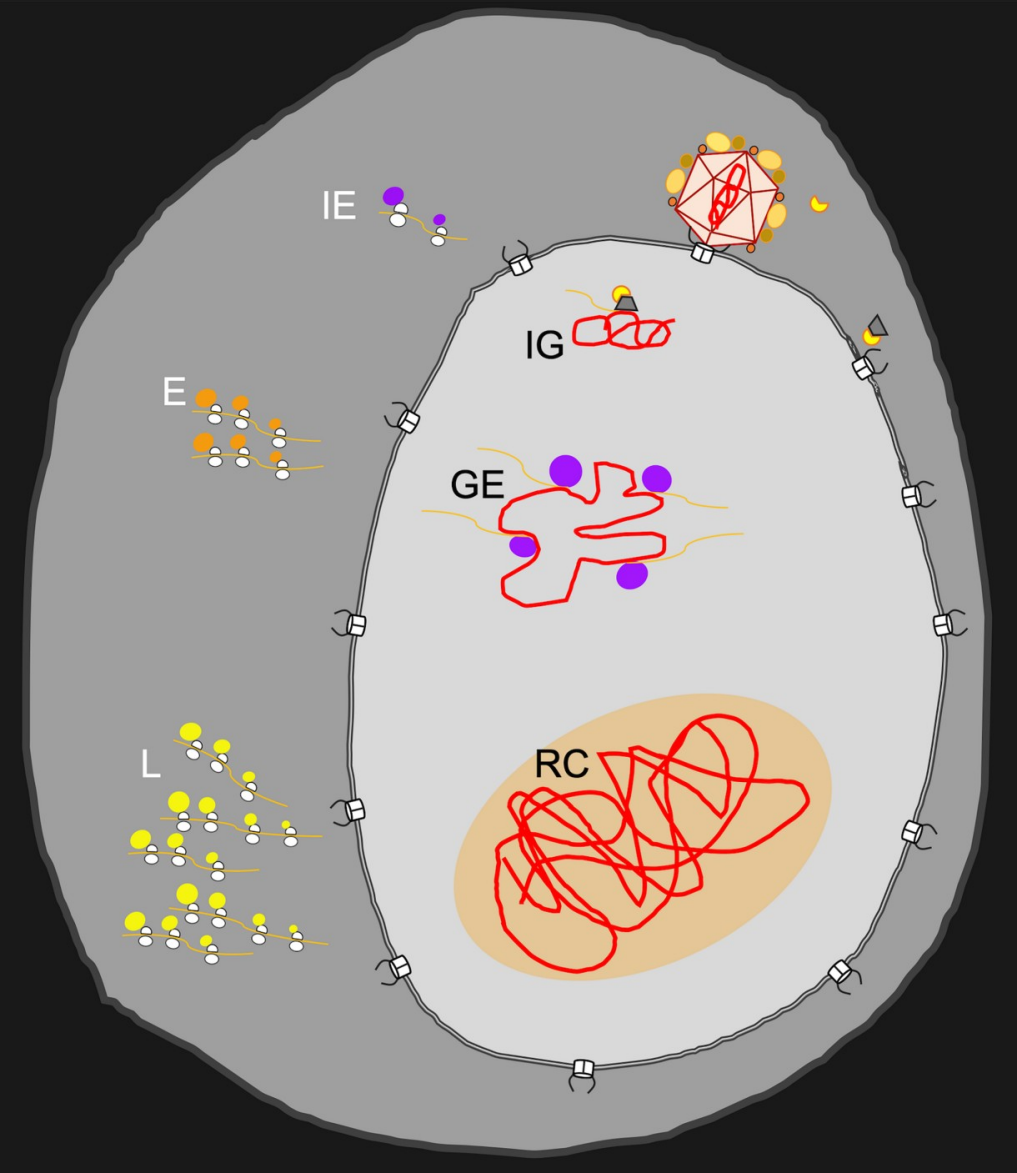
The cascade of events leading to RC formation. Schematic illustration of viral genomes entering the nucleus and forming RCs. Viral genome (red line) is released from the capsid at the nuclear pore complex. Once inside the nucleus, transcription from IG of IE genes (viral mRNAs presented as orange lines) is initiated by the VP16 complex (yellow and gray structure). Viral transcripts are exported to the cytoplasm for translation of IE (purple circles), E (orange circles), and L (yellow circles) proteins by host ribosomes. In the nucleus, IE proteins induce GE and E gene expression. E proteins establish viral DNA replication and L gene expression in RCs (RC shown as orange area in the nucleus). E, early; GE, genome expansion; IE, immediate early; IG, incoming genome; L, late; RC, replication compartment; VP16, viral protein 16.

**Fig 2:**
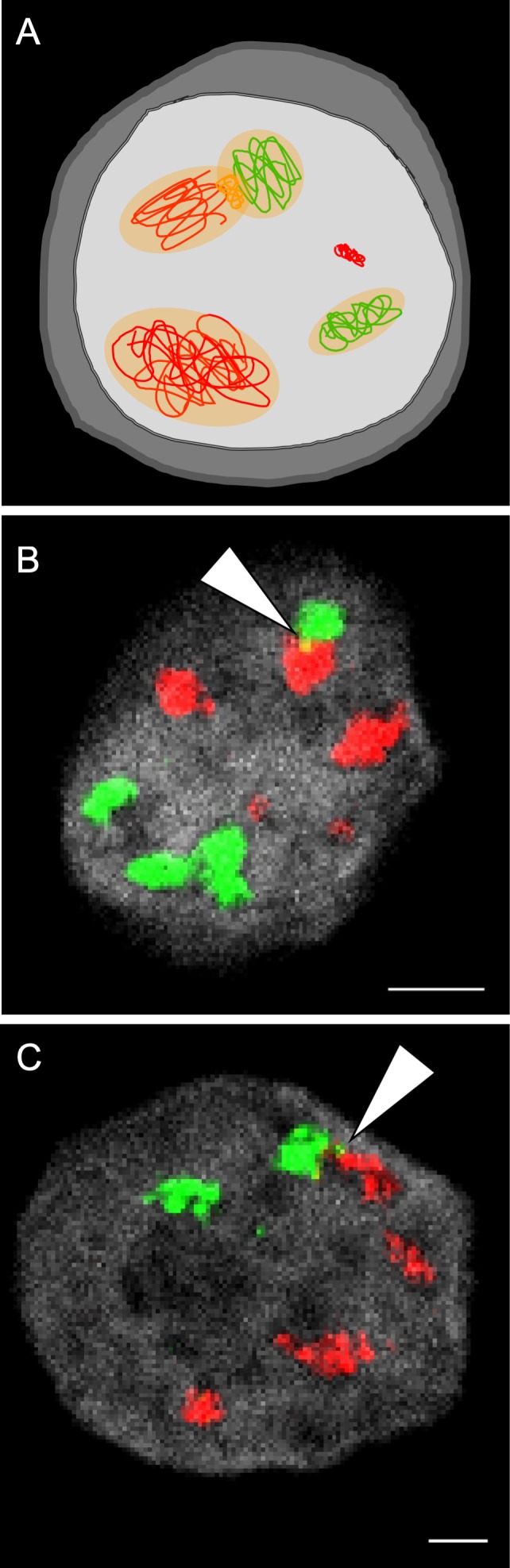
Heterogeneity and interactions among RCs. (A) Schematic illustration of an infected cell with two types of viral genomes (red or green lines) that form distinct RCs (RCs shown as orange area in the nucleus). Viral proteins within the RCs originate from the two genomes. Recombination events occur between two genomes in coalescing RCs (orange line). An entering genome that did not initiate an RC is also shown (condensed without background). (B, C) Fluorescence in situ hybridization image of U2OS (B) or Vero (C) cells 6 hours postinfection with two HSV-1 recombinants at MOI 20. Each viral recombinant carries a unique tag sequence that can be detected by a set of fluorescent probes (either green or red). Arrowhead points to a site of colocalization (indicating a possible recombination event). DNA staining was done by DAPI (gray). Scale bar, 20 μM. Experimental details were described [7]. MOI, multiplicity of infection; RC, replication compartment.

The HSV-1 genomes are packaged inside the viral capsid as a linear double-stranded DNA molecule that contains nicks and gaps [2]. These genomes enter the nucleus of infected cells through the nuclear pore complex as condensed naked DNA (Fig 1). Viral gene expression is proposed to be coupled with decondensation of the entering genomes [3]. Viral E proteins are then required for the onset of viral replication, turning sites of entering viral genomes into RCs (Fig 1). Recent studies detecting single incoming viral genomes using “click chemistry” revealed that each genome can form its own RC [3, 4]. Several different experimental approaches are also supportive of this hypothesis. Live-cell imaging showed that viral amplicons and helper virus replicate in distinct foci [5]. Using dual-color fluorescence in situ hybridization (FISH) for pseudorabies virus (PRV) [6] and HSV-1 [7], we showed that coinfecting herpesviruses’ RCs are mostly maintained in separate territories (Fig 2). Furthermore, a limited number of incoming herpes genomes initiate expression and replication within a given cell, and the number usually remains in the single-digit range [8, 9]. The average number of RCs per cell is also quantified within the same range [3, 10, 11]. Recently, we were able to demonstrate that cell types able to support replication of more incoming viral genomes show a higher number of RCs per cell [7]. Whereas the majority of RCs initiate from single incoming genomes, there is a small population of RCs that appear to emerge from more than one genome [7].

## How do RCs recruit viral and host proteins?

Many viral and host proteins are thought to assemble on viral genomes to form RCs. The RCs formed during HSV-1 infection were originally identified as nuclear sites containing viral DNA associated with viral single-stranded DNA–binding protein ICP8 and viral transcription activator ICP4 [12, 13]. RCs are the sites of viral DNA replication, and therefore, many host and viral proteins involved in replication, recombination, DNA repair, and viral L gene expression are recruited to the RCs. Recent proteomics approaches have used mass spectrometry to identify proteins associated with labeled viral DNA [3, 4, 14, 15]. Some proteins interact specifically with viral sequences, such as viral protein ICP4 and the viral protein 16 (VP16) complex [16, 17], whereas others are recruited via protein–protein interactions with DNA-binding proteins.

The nature of the viral DNA presumably contributes to protein recruitment. Viral DNA enters the nucleus as an open-ended molecule with nicks and gaps [2]. These features are likely to induce interactions with proteins involved in the cellular DNA damage response [4, 14, 18]. In addition, the incoming foreign DNA is bound by host intrinsic defense factors such as interferon gamma inducible protein 16 (IFI16) and components of promyelocytic leukemia protein (PML) nuclear bodies [19]. Association of host proteins with the viral genome is dynamically regulated during infection [4, 20], and the ubiquitin ligase activity of the viral ICP0 protein can remove inhibitory host factors [21]. The initial loading of histones onto the viral DNA is dependent on histone chaperone protein antisilencing function 1A histone chaperone (ASF1A) [22]. It was recently suggested that different mechanisms are employed to perform the initial chromatinization and the maintenance of histones on viral genomes [23]. However, during lytic replication, the viral DNA is found in the nucleus with relatively low abundance of host histones, providing the opportunity for other nonspecific DNA-binding proteins to accumulate [24].

Recent studies of subcellular compartments suggest a role for liquid phase separation as a way to sequester specific proteins within compartments that are not separated by membranes [25]. These structures are proposed to be formed by multivalent protein–protein interactions mediated by intrinsically disordered regions of proteins that weakly interact and can be promoted through binding nucleic acids [25]. HSV-1 RCs possess several characteristics of liquid phase separation such as spherical shapes, the ability to fuse, and enrichment of viral IE proteins with intrinsically disordered regions [24]. However, a recent study suggests that at least RNA polymerase II is incorporated into RCs by transient DNA binding and not via multivalent protein–protein interactions [24].

## What are the interactions between RCs?

Viral RCs are dynamic structures that change during infection with respect to their size, position in the nucleus, and protein composition. As replication proceeds, the size of the RC grows [3], and the likelihood of RCs merging in the limited space of the nucleus increases. It has been demonstrated that RCs move and coalesce by directed motion [10, 26]. This motion is stalled by inhibitors of actin, myosin, and transcription. Furthermore, RCs move toward nuclear speckles (sites of RNA processing) and coalesce near these sites [26].

Several observations support the possibility that viral proteins encoded in one RC can spread to all other RCs within the same nucleus. First, complementation between coinfecting herpesviruses (even between different viruses in the family) is well established. Furthermore, in PRV we found that L capsid proteins from different genomes are distributed evenly among the newly formed capsids [6]. This might not be the case at early stages of viral IE and E gene expression. It is possible that IE proteins that are needed to overcome host repression are not spread equally between entering genomes, since not all viral genomes in the nucleus have the same fate [3].

By detecting different viral genomes within a cell, we found that each genome maintains its own territory even at points of intersection between RCs [6, 7]. This suggests that mixing at the DNA level is limited between RCs. Because intergenomic recombination is a common phenomenon during HSV-1 coinfection and considered a major driving force of evolution in these viruses [27], the limited mixing of DNA raises questions about where recombination occurs. Our results suggest that recombination between coinfecting genomes is likely to arise among coalescing RCs at the sites of joining [7].

## Are all RCs created equal?

Imaging RCs has revealed that, within single cells, not all RCs are the same size and shape (Fig 2). At early time points, the sites of replication and transcription colocalize, but later during infection, sites dedicated only for replication appear [11]. It is likely that these are extensions of growing RCs, since the number of replication sites increases with infection time. The possible heterogeneity of RCs within a given cell raises additional questions. Do all RCs have similar protein content? Do all RCs have a similar capacity to generate viral progeny? Are specific functions assigned to distinct RCs? Do different RCs produce progeny viruses with different protein composition? These questions are currently technically challenging to address, but probing these issues could provide insights into host–virus interactions that are important for the formation of new progeny viruses.

## Conclusions

The distinct intranuclear structures formed during HSV-1 infection represent hubs for viral gene expression, replication, and encapsidation. Biochemical and imaging approaches have defined components of these viral-induced structures. Although these studies have helped to identify potential players in the process of viral replication, these findings raise many new questions about how these interactions impact the outcome of infection. Is there a competition between RCs for limited cellular resources? To what extent do viral RCs possess characteristics of liquid organelles? What is the significance of heterogeneity among RCs? Ongoing technology improvements will allow better understanding of the processes required for viral replication. Viral research has provided insights into many fundamental host mechanisms. Because viral replication is a rapid event that dramatically alters nuclear architecture, understanding mechanisms involved in RC formation, maintenance, and interactions will shed further light on structural processes and regulation within the mammalian nucleus.
